# Long-term efficacy of enucleation combined with primary orbital implantation in children with retinoblastoma histopathological invasion of optic nerve

**DOI:** 10.3389/fneur.2022.1013523

**Published:** 2022-10-10

**Authors:** Nan Wang, Rui Liu, Jing Li, Jinjin Wang, Liangyuan Xu, Qihan Guo, Xuan Zhang, Jianmin Ma

**Affiliations:** ^1^Beijing Ophthalmology and Visual Sciences Key Laboratory, Beijing Tongren Eye Center, Beijing Tongren Hospital, Capital Medical University, Beijing, China; ^2^Tai'an City Central Hospital, Qingdao University, Tai'an, China

**Keywords:** retinoblastoma, optic nerve invasion, enucleation, primary orbital implantation, long-term effect

## Abstract

**Objective:**

This study aimed to observe the long-term effect of enucleation combined with primary orbital implantation in children with histopathologic optic nerve invasive retinoblastoma (RB).

**Methods:**

We retrospectively analyzed the clinical data and outcomes of children with RB optic nerve invasion confirmed by histopathology who underwent enucleation combined with primary orbital implantation between March 2010 and April 2014 in Beijing Tongren Hospital. The follow-up time ranged from 81 to 129 months, with a mean follow-up of 96 ± 14 months.

**Results:**

A total of 59 children were included in this study. There were 32 males and 27 females; 52 children were affected in one eye and seven children in both eyes. The time from onset of symptoms to visit was between 3 days and 16 months, with a mean of 2.2 ± 2.8 months. The age at the of surgery was between 2 and 65 months, with an average of 24 ± 13 months. Patients were classified based on the degree of optic nerve invasion into four grades: grade 1 (invasion of prelaminar) in 28 cases, grade 2 (invasion of laminar) in 14 cases, grade 3 (invasion of retrolaminar but not reaching the optic nerve transection) in 16 cases, and grade 4 (invasion of the optic nerve transection) in 1 case. Post-operatively, all children were treated with 0–9 cycles of intravenous chemotherapy based on histopathology results from the removed eye. Of the 59 children, 13 had postoperative complications, and one died from recurrence. The survival rate was 98% (58/59). There was one case of orbital implant exposure, one of orbital cellulitis, six of enophthalmos and superior sulcus deformity, two of blepharoptosis, one of granuloma complicated with blepharoptosis, and one with a subconjunctival cyst.

**Conclusion:**

For children with RB histopathologic invasion of the optic nerve, enucleation combined with primary orbital implantation reveals future potential treatment options when combined with a full course of intravenous chemotherapy.

## Introduction

Retinoblastoma (RB) is the most common primary intraocular malignancy in childhood (1). Metastasis of retinoblastoma occurs most often along the optic nerve to the central nervous system and is a leading cause of death in children (2). Therefore, optic nerve invasion of RB, especially retrolaminar tumor infiltration, is considered a poor prognostic sign, associated with an increased possibility of metastasis (3).

The development of RB comprehensive treatment concept has significantly improved the long-term survival rates of patients (4). Therefore, the focus of treatment has shifted to improving the quality of life. For children with advanced RB, primary enucleation provided a survival advantage over eye salvage attempts. However, the normal development of orbit depends on stimulation from the globe (5). The loss of the globe from enucleation may lead to delayed craniofacial development and orbital deformity, both of which require stimulation from it (6). At present, implants are used to fill the orbital space and stimulate the normal development of adjacent bones to prevent deformity. However, at present, there is no international agreement or consistent gold standard for enucleation with or without orbital implant for retinoblastoma (5). This study analyzed the clinical results of unilateral enucleation combined with primary orbital implantation in children with RB and optic nerve invasion to observe and evaluate its safety, therapeutic effect, and complications.

## Research object and method

### Research object

We collected the clinical results of children with RB admitted to the Department of Ophthalmology and Oncology, Beijing Tongren Hospital, Affiliated with Capital Medical University, from March 2010 to April 2014. Inclusion criteria were as follows. (1) Combined with clinical manifestations, a diagnosis of RB based on Rectcam fundus imaging, orbital CT, and MRI. (2) No evidence of metastasis prior to surgery. (3) Classified as International Intraocular Retinoblastoma Classification (IIRC) stage D or E, and any conservative treatment failed. (4) An enucleation with primary orbital implantation was performed. (5) After enucleation, the optic nerve had RB confirmed by histopathology.

The exclusion criteria were as follows. (1) RB patients with pre-operative evidence of imaging detectable mss that invade the optic nerve and orbital or metastasis. (2) They had trilateral RB. (3) Patients who did not receive standardized treatment and follow-up according to their diagnosis and treatment plan. Written informed consent forms were signed by the guardians of all patients.

### Surgical methods

Under general anesthesia, the surgical area was disinfected. Next, the bulbar conjunctiva was cut along the corneal limbus, and the four rectus muscles were identified. A double-ring suture was set on the rectus tendon, and then rectus was separated and fixed while oblique muscles was not reserved. Next, the surrounding tissues were separated, and the optic nerve was cut for removal of the globe for histopathology. And the optic nerve resection length was 10 mm or more. A steel ball of the appropriate size was used to compress and stop the bleeding. Next, the hydroxyapatite implant (Bio-Eye OH Patite Orbital Implant, Beijing, China, China) was placed into the eye socket, and the sterile allogeneic sclera (provided by Beijing Tongren Hospital, Affiliated to Capital Medical University) spread on the front surface of it. Then sutured the four rectus to the position close to the center of the allogeneic sclera and the surgical incision sewn.

### Grade of optic nerve invasion

The histopathology results of the globe after enucleation were divided into four grades based on the extent of tumor invasion into the optic nerve. The grades were: grade 1 (invasion of prelaminar), grade 2 (invasion of laminar), grade 3 (invasion of retrolaminar, but not reaching the optic nerve transection), and grade 4 (invasion of the optic nerve transection).

### Postoperative chemotherapy

Chemotherapy is not recommended for children with RB invasion of the optic disc and lamina without other high-risk factors for metastasis. The grade 3 patients were treated with at least 6 cycles of intravenous chemotherapy, including vincristine, carboplatin, etoposide. The grade 4 patient were treated with for 9–12 cycles of intravenous chemotherapy and intrathecal chemotherapy (cytarabine, methotrexate, and dexamethasone).

### Follow-up

All children were followed up through outpatient, telephone, or WeChat visits. Prognostic information was collected and summarized.

### Statistical analysis

SPSS 25.0 and Graphpad Prism 8.0 statistical software were used to perform the analysis. The measurement data were tested using a *t*-test, and the counting data were analyzed with a Chi-square test. The Kaplan-Meier survival curves were used to estimate overall survival, and the subgroups were compared using a log-rank test (Mantel-Cox). A *p*-value < 0.05 was considered statistically significant.

## Results

**Figure 1:**
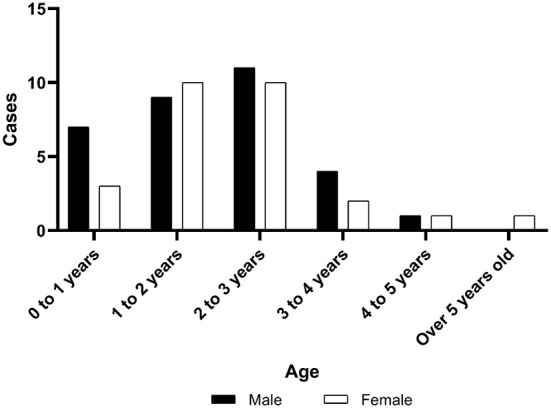
Gender distribution of children in different age groups.

A total of 59 children were included in the study. There were 32 males and 27 females; 31 had RB in the right eye and 28 in the left; 52 (88%) unilateral and 7 (12%) bilateral. The ages ranged from 2 to 65 months, with an average of 24.00 ± 13.35. Among them, seven children (7/59, 11.9%) were younger than 6 months at the time of surgery. The IIRC stages indicated 28 cases in stage D and 31 in stage E. The time between the onset of symptoms to visit ranged from 3 days to 16 months, with an average of 2.2 ± 2.8 months (Figure 1).

The chief complaint for most children was leukocoria (37/59, 63%), while others complained of red eyes (8/59,14%), strabismus (4/59, 6.8%), reduced vision (4/59,6.8%), photophobia and lacrimation (3/59,5.1%), exophthalmos (1/59,1.7%), corneal opacity (1/59,1.7%), or mydriasis (1/59,1.7%). Preoperative examination of the fundus with a Retcam or ultrasound showed retinal detachment in 27 children (46%).

Before enucleation, 12 children underwent intravenous chemotherapy; 2 children underwent intravenous chemotherapy combined with photocoagulation; 1 child underwent photocoagulation; 1 child underwent cryotherapy; and 42 children had no treatment.

The postoperative histopathological results indicated optic nerve invasion was grade 1 in 27 cases (46%), grade 2 in 14 (24%), grade 3 in 17 (29%), and grade 4 in 1 (1.7%). In addition, to optic nerve invasion, 14 children had it combined with other histopathological high-risk factors, including 7 cases of massive choroid invasion (>3 mm), 5 cases of anterior chamber invasion, and 2 cases of massive choroid and anterior chamber invasion simultaneously. The probability of optic nerve invasion combined with other high-risk factors for metastasis was 11% (3/27) in grade 1, 29% (4/14) in grade 2, 35% (6/17) in grade 3, and 100% (1/1) in grade 4. The average age of those with histopathological high-risk factors was 29 ± 13 months, which was higher than those without (20 ± 12 months, *p* = 0.08). A total of 39.3% (11/28) of stage D children had high-risk factors for metastasis, which was lower than those with stage E (45%, 14/30) (*p* = 0.65). Clinical features of 59 children with optic nerve invasion RB show in Table 1.

**Table 1:** Clinical features of 59 children with optic nerve invasion RB.

	**Number of cases**	**Percentage (%)**
**Gender**		
Male	32	54
Female	27	46
**Unilateral or bilateral**		
Bilateral	7	12
Unilateral	52	88
**Disease laterality**		
Right	31	53
Left	28	47
**Disease stage**		
Stage D	28	47
Stage E	31	53
**Size of orbital implant**		
18 mm	13	22
20 mm	28	47
22 mm	18	31
**Other treatments**		
**Preoperative**		
Intravenous chemotherapy	12	20
Intravenous chemotherapy and photocoagulation	2	3.4
Photocoagulation	2	3.4
Cryotherapy	1	1.7
**Postoperative**		
Intravenous chemotherapy	39	51
Intravenous chemotherapy and radiation therapy	1	1.7
**Grade of optic nerve invasion**		
Grade 1	27	46
Grade 2	14	24
Grade 3	17	29
Grade 4	1	1.7

After surgery, 39 children also underwent intravenous chemotherapy, while 20 did not. Of those that had intravenous chemotherapy, 10 had retrolaminar optic nerve invasion. Another three cases had simultaneous invasion of the optic nerve and choroid. Additionally, 14 cases were combined with other high-risk metastasis factors including 5 cases with invasion of the anterior chamber, 7 were massive choroid (>3 mm) invasion, and 2 had simultaneous invasion of the anterior chamber and a massive choroid. Other cases included four that required chemotherapy due to bilateral disease, while parents of eight more cases requested chemotherapy. Only 1 patient was treated with 9 cycles of intravenous chemotherapy combined with 22 rounds of local radiotherapy after surgery. This patient also had enophthalmos and superior sulcus deformity 4 years after surgery but had no tumor recurrence during follow-up.

**Figure 2:**
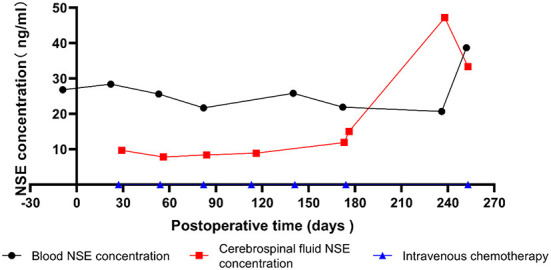
Levels of neuron specific enolase (NSE) over time in patient that died.

The follow-up time ranged from 81 to 129 months, with an average of 96 ± 14 months. The overall survival rate was 98% (58/59). The one patient that died resulted from recurrence and intracranial metastasis 8 months after surgery. His clinical characteristics show in Table 2. The fundus examination showed secondary glaucoma, corneal opacity, hyphema, mydriasis, and an unknown fundus. Histopathological results showed a grade 3 optic nerve invasion. The tumor involved massive choroid, anterior chamber, anterior chamber angle and iris, and had high-risk factors for metastasis. Six cycles of regular chemotherapy were given after surgery. Eight months after surgery, the child developed intermittent headaches with vomiting, they had a significant increase in cerebrospinal fluid neuron-specific enolase (NSE) (Figure 2), and MRI showed a mass shadow in the left orbit, which was considered tumor recurrence.

**Table 2:** Clinical characteristics of patient that died.

Age (months)	28
Staging (ICR staging)	Stage E
Chief complaint	Red eyes
Curse of disease (months)	2
Size of orbital implant (mm)	22
Preoperative treatment	None
Postoperative chemotherapy	7 cycles
High-risk histopathologic features	The tumor invaded retrolaminar optic nerve, but did not reach the optic nerve transection; invasion of the massive choroid, anterior chamber angle, and iris stroma; grew close to corneal endothelium and ciliary body surface.
Time to recurrence	8 months after surgery

**Figure 3:**
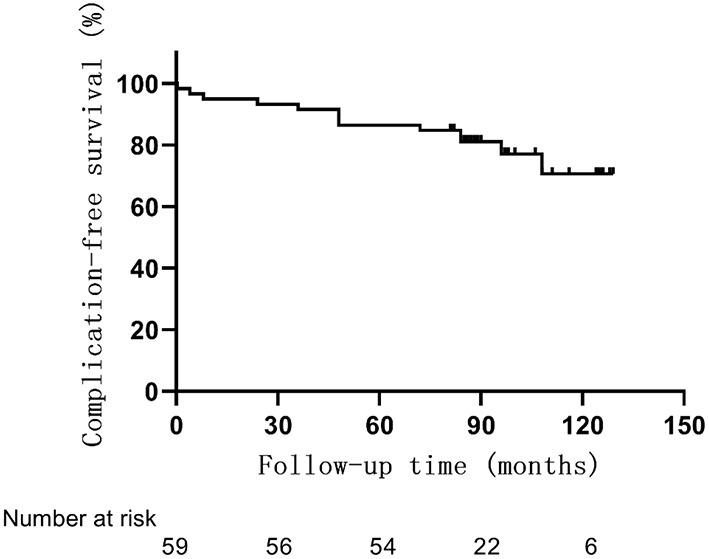
Overall rate of complications in 59 children with RB optic nerve invasion.

Postoperative complications occurred in 13 patients (22%) (Table 3, Figure 3). One patient had orbital implant exposure on the 10th day after surgery. The child underwent orbital implant exposure repair and recovered well. Another patient developed orbital cellulitis 4 months after surgery but improved with treatment with anti-inflammatory and anti-infective therapy. Two additional children developed blepharoptosis at 4 or 7 years after surgery. Another child developed a granuloma 9 years after surgery and blepharoptosis 10 years after enucleation. Yet another child developed a subconjunctival cyst 2 years after surgery. Six more children had enophthalmos and superior sulcus deformity, four of whom had an 18 mm orbital implant and two that had a 20 mm orbital implant.

**Table 3:** Complications of enucleation combined with primary orbital implantation in children with RB optic nerve invasion.

**Complications**	**Cases (%)**	**Time to occurrence of complications (years)**
		**Average (range)**
Metastasis and death	1 (1.7)	0.67 (0.67)
Orbital implant exposure	1 (1.7)	0.03 (0.03)
Orbital cellulitis	1 (1.7)	0.33 (0.33)
Enophthalmos and superior sulcus deformity	6 (10)	5.33 (3–8)
Blepharoptosis	3 (5.1) (one with Granuloma)	7.00 (4–10)
Subconjunctival cyst	1 (1.7)	2 (2)
Granuloma	1 (1.7)	9 (9)

**Figure 4:**
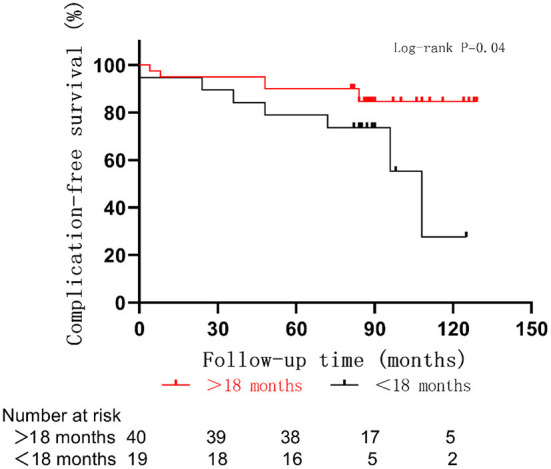
Rate of complications by age group in 59 children with RB optic nerve invasion.

**Figure 5:**
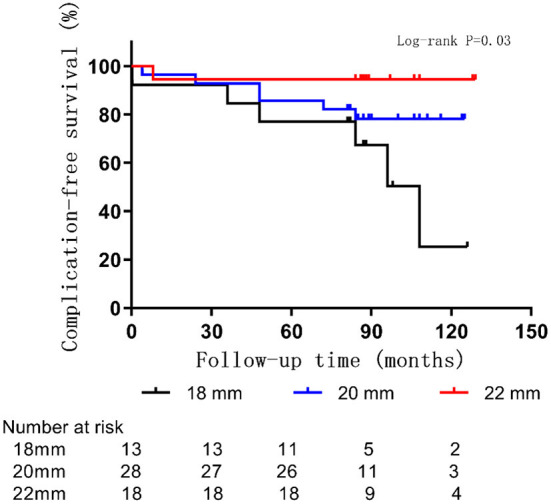
Rate of complications by orbital implant size in 59 children with RB optic nerve invasion.

**Figure 6:**
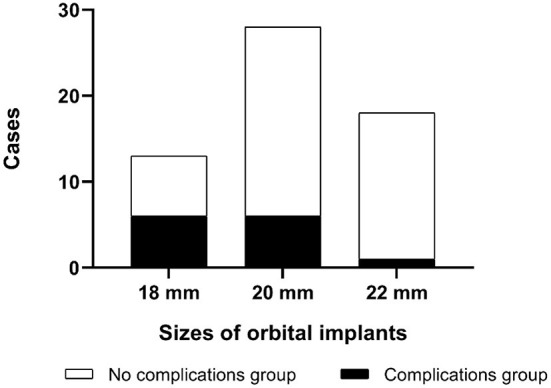
Complications in RB optic nerve invasion of patients with different sizes of orbital implants.

**Figure 7:**
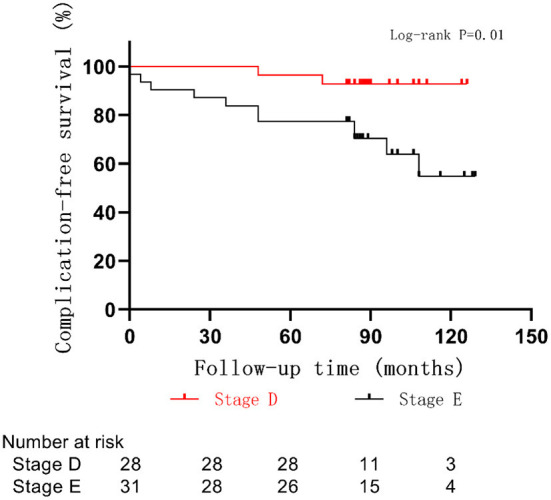
Rate of complications by state in of 59 children with RB optic nerve invasion.

The average age at the time of surgery of children with postoperative complications was 18 ± 13 months, which was lower than that of those without postoperative complications (26 ± 13 months, *p* = 0.06) (Figure 4). The complication rate of children under 6 months was 43% (3/7), which was higher than that of children over 6 months (19%, *p* = 0.35). A correlation between smaller-sized orbital implants and postoperative complications was seen. The probability of postoperative complications with an 18 mm orbital implant was 46% (6/13), while a 20 mm one had 21% (6/28), and those with a 22 mm one was 5.6% (1/18) (*p* = 0.03) (Figures 5 and 6). The pairwise comparison revealed a statistically significant difference between the 18 mm group and the 22 mm one (*p* = 0.01), while the other groupings were not (18 mm and 20 mm, *p* = 0.15; 20 mm and 22 mm, *p* = 0.22). The probability of complications in stage D was 7.1% (2/28), and in stage E was 36% (11/31) (Figure 7). The difference was statistically significant (*p* = 0.01). The average course of disease in children with complications was 3.5 ± 4.7 months, which was longer than without (1.9 ± 1.9 months, *p* = 0.06). Other clinical characteristics included gender (male 19%, female 26%, *p* = 0.54), disease location (right eye 19%, left eye 25%, *p* = 0.76), unilateral or bilateral (unilateral 21%, bilateral 29%, *p* = 0.64), presence of retinal detachment before surgery (presence 22%, absence 22%, *p* = 0.97), occurrence of high-risk histopathologic features (with 20%, without 24%, *p* = 0.75), presence of retrolaminar optic nerve invasion (with 17%, without 24%, *p* = 0.51), and need for postoperative chemotherapy (26% for with and 15% without, *p* = 0.35) all had no significant effect on the occurrence of postoperative complications. Influencing factors of enucleation complications show in Table 4.

**Table 4:** Influencing factors of enucleation complications in children with RB optic nerve invasion.

	**No complication group, *n* (%*n*)**	**Complication group, *n* (%*n*)**	***p*-value**
Gender			0.54
Male	26 (81)	6 (19)	
Female	20 (74)	7 (26)	
Average age (months)	20 ± 12	29 ± 13	0.06
Mean course of disease (months)	1.9 ± 1.9	3.5 ± 4.7	0.06
Unilateral or bilateral			0.64
Bilateral	5 (71)	2 (29)	
Unilateral	41 (79)	11 (21)	
Disease laterality			0.76
Right	25 (81)	6 (19)	
Left	21 (75)	7 (25)	
Disease stage			0.01
Stage D	26 (93)	2 (7)	
Stage E	20 (65)	11 (35)	
Size of orbital implant			0.03
18 mm	7 (54)	6 (46)	
20 mm	22 (79)	6 (21)	
22 mm	17 (94)	1 (6)	
Retinal detachment before surgery			0.97
No	25 (78)	7 (22)	
Yes	21 (78)	6 (22)	
Other high-risk histopathologic features			0.75
No	26 (76)	8 (24)	
Yes	20 (80)	5 (20)	
Postoperative chemotherapy			0.35
No	17 (85)	3 (15)	
Yes	29 (74)	10 (26)	
Retrolaminar optic never invasion			0.51
No	31 (76)	10 (24)	
Yes	15 (83)	3 (17)	

## Discussion

RB is the most common intraocular malignant tumor in childhood, and according to the Thai Pediatric Oncology Group (ThaiPOG), it has surpassed uveal melanoma to become the most common ocular malignant tumor globally (7). Although the incidence of metastasis is quite low, the most common metastatic site of RB is central nervous system, accounting for about 50% of total metastases, and causes the most deaths in children (3). Ren et al. (8) found that tumors adjacent to the optic disc may spread intracranially through the optic nerve resulting in central nervous system metastasis. Also, retrolaminar invasion indicates that the tumor is extraocular, which has high metastasis and poor prognosis. The greater the degree of optic nerve invasion, the lower the incidence, while metastasis and mortality increase. The mortality rates of patients in grades 1 to 4 were 0.4–10, 1.0–29, 8.7–42, and 60.9–78%, respectively (9, 10). Patients with retrolaminar optic nerve invasion have a statistically higher risk of metastasis, and the mortality rate is as high as 50%−81% in children with positive optic nerve margins (11, 12). Therefore, retrolaminar optic nerve invasion has a poor prognosis and is associated with local recurrence and systemic metastasis (5, 13). Most studies recommend preoperative orbital and brain/spine MRI, cerebrospinal fluid cytology, and fundus examination to confirm clinical staging and metastasis (14). Based on findings, chemotherapy is used to reduce the tumor size, allowing for enucleation, followed by postoperative chemotherapy and radiotherapy according to the histopathology results (12). However, weighing the benefits against the side effects in recent studies has resulted in the stopping of prophylactic radiotherapy (15).

RB treatment has been for survival first, followed by globe and vision preservation. However, with the development of a comprehensive treatment concept and the significant improvement of survival rate, for children undergoing enucleation, a fourth treatment goal is to restore facial features and enhance the quality of life (3). This is important since most patients are under 5 years old (16). After enucleation, the lack of the globe results in poor development of orbital bones that leads to orbit and facial deformities and physical and psychological trauma.

However, at present, there is no international agreement or consistent gold standard for enucleation with or without orbital implant for retinoblastoma. Up to now, no research has clearly proved the safety, especially the long-term impact of primary orbital implantation of histopathologic optic nerve invasion patients (5). In the observation of optic nerve invasion, imaging examination always lags behind histopathologic changes. We observed patients who showed no obvious mass of optic nerve on imaging examination, but detected histopathologic optic nerve invasion to determine whether enucleation combined with primary orbital implantation is safe and does not increase the risk of recurrence and metastasis. In this study, 59 childrenwere followed for ~8 years, only one case of intraorbital metastasis occurred, resulting in a survival rate of 98%. These findings indicate that RB patients with histopathologic optic nerve invasion can undergo primary orbital implantation with adequate intravenous chemotherapy and have a good survival rate. The only death in this study occurred in a stage E patient with secondary glaucoma. His histopathology result showed the tumor involved optic nerve, massive choroid, anterior chamber, and had high-risk factors for metastasis. The RB recurred within 2 months after the postoperative chemotherapy was interrupted. Therefore, close observation with regular intravenous chemotherapy must be performed to monitor and prevent a recurrence.

Previous studies have suggested that for patients with grade 1 or 2 optic nerve invasion, postoperative intravenous chemotherapy is unnecessary. In comparison, postoperative intravenous chemotherapy is required for patients with grade 3 optic nerve tumor invasion or who have high-risk histopathologic factors. Patients with optic nerve transection involvement should receive high-dose systemic chemotherapy and orbital radiation therapy (10, 11). In this study, long-term observation of patients with grade 1 and 2 optic nerve invasion without other high-risk histopathologic features indicated no tumor recurrence with treatment options of primary orbital implantation without systemic chemotherapy. For those with retrolaminar optic nerve invasion, a high survival rate (94%) can still be obtained after enucleation when combined with primary orbital implantation and adequate intravenous chemotherapy. Therefore, for those with histopathologic optic nerve invasion, primary orbital implantation does not increase the risk of recurrence or affect the survival rate with adequate systemic chemotherapy.

Orbital implant exposure and orbital cellulitis are serious postoperative complications, but the incidence of them is low (5.1%) and occurs within 1 year of surgery. Most postoperative complications in this study were minor and affected facial appearance, including enophthalmos, superior sulcus deformity, blepharoptosis, granuloma, or subconjunctival cyst, which occurred 2 years after surgery. A young age at the time of surgery, a smaller-sized orbital implant, a longer course of the disease, or an advanced stage indicated a higher probability of postoperative complications. The size of the orbital implant was fixed. At the same time, the orbit volume continued to grow with age resulting in a diminishing of the stimulating effect from the implant over time. As a result of failing to balance the two, a lag in orbital bone development occurred compared to the contralateral side. It is also possible that the orbital implant compresses the adjacent adipose tissue causing atrophy and resulting in enophthalmos and superior sulcus deformity. Therefore, these complications may be directly related to the young age and small-sized orbital implants. Size selection becomes difficult as too small may reduce normal orbit development due to insufficient mechanical stimulation. However, if the orbital implant is too large, it will increase the difficulty of orbital implantation, result in poor healing of the postoperative conjunctival incision, or result in exposure and emergence of the orbital implant.

A large retrospective study reported a 4.2% (71/1674) incidence of orbital recurrence after enucleation, indicating a relatively rare complication (17). In this study, enucleation combined with primary orbital implantation was performed on children with histologically confirmed optic nerve invasion with RB. After sufficient treatment with intravenous chemotherapy, only one death from recurrence happened, and there was no increase in the recurrence or the long-term survival rates. Advantages of primary orbital implantation as compared with secondary orbital implantation include (1) avoidance of postoperative adhesions and contractures that may cause difficulties or unsatisfactory results with secondary orbital implantation; (2) stimulation of the craniofacial development earlier and in a timelier manner; (3) better restoration of the facial appearance; (4) avoidance of pain from multiple surgeries; and (5) reduced financial burden on families.

In summary, there was no evidence that adequate intravenous chemotherapy and enucleation combined with primary orbital implantation significantly increased the recurrence rate in RB patients with optic nerve invasion. The treatment appears safe and had few complications, all while avoiding facial developmental abnormalities caused by enucleation.

## Data availability statement

The original contributions presented in the study are included in the article/supplementary material, further inquiries can be directed to the corresponding author.

## Ethics statement

Ethical review and approval was not required for the study on human participants in accordance with the local legislation and institutional requirements. Written informed consent to participate in this study was provided by the participants' legal guardian/next of kin.

## Author contributions

NW analyzed and wrote the manuscript. JW, LX, XZ, and QG helped collect data. J-mM, JL, and RL read and criticized the manuscript. All authors critically read and edited the manuscript and read and approved the final manuscript.

## Funding

This work was Supported by Natural Science Foundation of Beijing (7222025) and Beijing Hospitals Authority' Ascent Plan (DFL20190201).

## Conflict of interest

The authors declare that the research was conducted in the absence of any commercial or financial relationships that could be construed as a potential conflict of interest.

## Publisher's note

All claims expressed in this article are solely those of the authors and do not necessarily represent those of their affiliated organizations, or those of the publisher, the editors and the reviewers. Any product that may be evaluated in this article, or claim that may be made by its manufacturer, is not guaranteed or endorsed by the publisher.

## Abbreviations

RB: retinoblastoma
IIRC: International Intraocular Retinoblastoma Classification
NSE: neuron-specific enolase.
